# Protocol for *in vivo* analysis of pre- and post-synaptic protein function in mice

**DOI:** 10.1016/j.xpro.2024.103117

**Published:** 2024-06-09

**Authors:** Teresa M.L. Cramer, Andrin Abegg, Shiva K. Tyagarajan

**Affiliations:** 1 University of Zurich, Institute of Pharmacology and Toxicology, Winterthurerstrasse 190, 8057 Zurich, Switzerland

**Keywords:** cell biology, microscopy, neuroscience

## Abstract

Studying synapses *in vivo* presents challenges due to the complexity of accurately targeting and visualizing specific synaptic proteins within the brain. Here, we present a protocol for *in vivo* analysis of pre- and post-synaptic protein function in mice. We describe steps for combining adeno-associated virus (AAV)-mediated gene transfer to manipulate specific neuron subtypes. We also describe immunofluorescence on artificial cerebrospinal fluid (ACSF)-perfused brain sections to enhance the visualization of synaptic proteins.

For complete details on the use and execution of this protocol, please refer to Cramer et al.^1^

## Highlights

• ACSF perfusion technique for investigating synaptic proteins *in vivo*
• Steps for AAV gene transfer for broad expression or in specific neurons
• Instructions for immunofluorescent labeling of free-floating brain sections
• Guidance on improved detection of synaptic proteins using microscopy

## Abstract (editor highlights)

Publisher’s note: Undertaking any experimental protocol requires adherence to local institutional guidelines for laboratory safety and ethics.

## Graphical abstract

**Figure:**
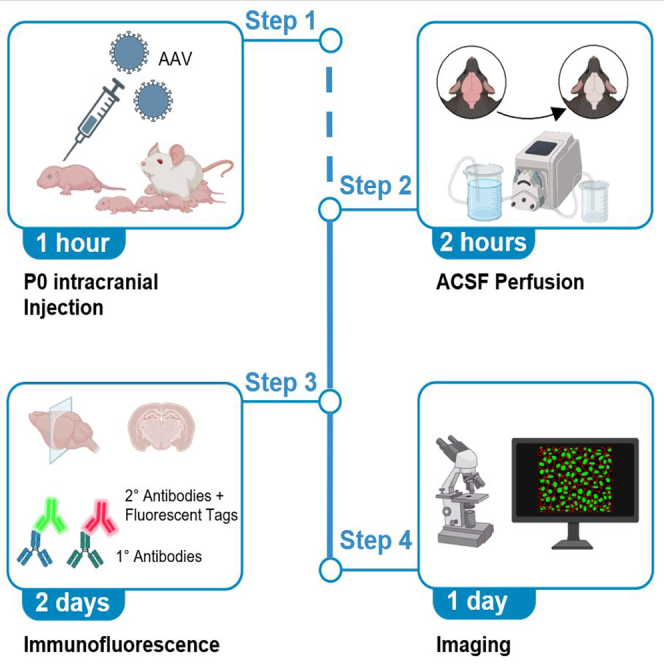

## Before you begin

This protocol describes a method to investigate the function of synaptic proteins *in vivo* using a combination of adeno-associated virus (AAV)-mediated gene transfer and immunofluorescence. To achieve long-lasting expression of transgenes, we describe a simple technique involving neonatal intraventricular injection of genetically engineered AAVs. Compared to the common route of AAV delivery through stereotaxic surgeries, AAV injections into the neonatal ventricles are more time efficient and achieve widespread transgene expression^2^^,^^3^ at the expense of spatial specificity. The resultant expression of transgenes can further be specified by the use of distinct promoters, providing an easy way to manipulate specific neuronal subtypes and their synapses throughout the brain. Particularly valuable for the investigation of synapses is the segregated manipulation of pre-synaptic or post-synaptic neurons, as it enables the investigation of pre- or post-synaptically localized proteins, as well as the site-specific function of secreted proteins localized in the synaptic cleft. To further study their function, immunofluorescence is commonly used, which enables the visualization of highly specific protein targets present at synapses using a combination of primary and secondary antibodies. Despite being a powerful technique, its effectiveness is often limited by tissue preparation methods employing transcardiac perfusion with 4% PFA, as the strength of this fixation method severely limits the immunoreactivity and detection of proteins in numerous subcellular compartments, particularly at synaptic sites. As reliable detection of synaptic proteins is crucial to understanding their function, this protocol also describes the ACSF perfusion method, which reduces the strength of tissue fixation and offers superior detection of pre- and post-synaptic proteins. This protocol is based on an initial publication investigating the function of Adamtsl3 at pre- and post-synaptic inhibitory sites using *Adamtsl3*^*flox/flox*^ mice described in Cramer et al., 2023.^1^ The presented protocol, however, can easily be applied in various settings. Before you begin, you will need to complete the following preparatory steps.

### Institutional permissions

All the procedures in animal studies must be approved by the Institutional Animal Care and Use Committee according to established regulations and guidelines. In this protocol, all procedures were reviewed by the European Community Council Directives of November 24, 1986 (86/609/EEC) and approved by the cantonal veterinary office of Zurich.

### Preparation one

#### Adeno-associated virus (AAV) selection

Recombinant AAV vectors are great tools for gene transfer and have become readily available. To achieve long-term expression of a protein of interest, the following considerations should be made when selecting an AAV.

1. The relative transduction efficiency of AAV serotypes should be considered when widespread expression is desired.^2^^,^^3^ We have observed efficient and widespread transduction in neonatal mouse brains from the hybrid serotypes 2/6, 2/8, and 2/9, but other serotypes may be suitable, and their transduction efficiencies should be evaluated before beginning your experiment. AAVs are limited by a relatively small genome size, which allows for an insert of approximately 4.7 kb. This limits their use for large or multiple payloads. Multiple payloads will often have to be delivered using multiple AAVs, which increases experimental complexity and reduces efficiency due to incomplete co-transduction. Oversized genomes containing inserts larger than 4.7 kb can be packaged, but the efficiency of the resulting AAV will be significantly reduced.
2. Nervous cell tropism varies among AAV serotypes and should be considered when cell type-specific transduction is desired. The 2/6, 2/8, and 2/9 serotypes provide excellent transduction *in vivo*. In addition, cell type-specific promoters should be utilized if astrocyte-, oligodendrocyte-, or neuron-specific transduction is desired, as well as the use of promoters for neuron subtype-specific transduction (excitatory, inhibitory, or exclusively parvalbumin-positive neurons for example).
3. Depending on the experimental aim, constitutive or inducible transgene expression should be considered. Constitutive transgene expression is essential when investigating early periods of development, while inducible transgene expression is often desired when protein function in the adult brain is investigated and developmental defects resulting from constitute transgene expression must be avoided. Among the inducible systems, the tamoxifen (TAM)-inducible system is the most widely used, enabling temporal control over transgene expression. Also depending on the brain region/synapse of interest, a combination of AAVs should be considered when pre-synaptic or post-synaptic neuron transduction is desired. For those experiments, both neuron subtypes should be transduced simultaneously in a separate cohort to confirm the effects of neuron subtype-specific transduction.

In this protocol, we transduced neonatal *Adamtsl3*^*flxo/flox*^ mice with AAV2/6-CaMKIIα-Cre-ERT2 or AAV2/8-hDlx-Cre-ERT2 to ablate *Adamtsl3* in adult mice either within pyramidal neurons or inhibitory interneurons respectively. On account of the well-described subcellular distribution of inhibitory synapses in the hippocampus, this combination enabled the selective investigation of presynaptic (hDlx-ERT2-Cre) and postsynaptic (CaMKIIα-Cre-ERT2) Adamtsl3 function at GABAergic synapses at CA1 pyramidal cell somata. We also injected a separate cohort with AAV2/8- hSyn1-Cre-ERT2 (not discussed) to achieve widespread transduction of all neurons to confirm the effects of neuron subtype-specific *Adamtsl3* ablation.

### Preparation two

#### Generation of neonatal pups for AAV injection

Intraventricular injections of genetically engineered AAV should be performed at post-natal day 0 (P0). To maximize the number of pups born for injection, it is beneficial to pair two female mice with one male mouse and set up 2 or more bleedings in parallel. Additionally, it is recommended to check the female mouse for the presence of a vaginal plug one day after mating. This plug serves as an indicator of successful mating and allows for estimation of the birth date as females will typically deliver 19 ± 1 day after the appearance of the plug. Three days prior to delivery, it is also advantageous to place the pregnant females into a clean cage to avoid males cannibalizing the newborn pups. In this protocol, we crossed mice to obtain *Adamtsl3*^*flxo/flox*^ pups and performed intraventricular injections within 12 h of birth.

## Key resources table

**Table:** 

REAGENT or RESOURCE	SOURCE	IDENTIFIER
**Antibodies**		

Rabbit polyclonal anti-Cre	BioLegend	Cat no. PRB-106P
Guinea pig polyclonal anti-GABA_A_R γ2 subunit	Home-made	Fritschy et al., 1992 https://doi.org/10.1073/pnas.89.15.6726
Mouse monoclonal anti-Gephyrin	Synaptic Systems	Cat no. 147111
Rabbit polyclonal anti-GABA_A_R α2 subunit	Synaptic Systems	Cat no. 224103
Rabbit monoclonal anti-VGAT	Synaptic Systems	Cat no. 131 003

**Other**		

Sliding Microtome Microm HM 400 R		
CLSM 800 Airyscan microscope, Carl Zeiss		

**Bacterial and virus strains**		

AAV8/2-hDlx-ERT2-Cre	Viral Vector Facility, UZH/ETH	Cat no. v301
AAV6/2-CaMKIIα-ERT2-Cre	Vector Biolabs	Cat no. 2014-1208

**Chemicals, peptides, and recombinant proteins**		

10 μL Hamilton syringe with 32 gauge needle	Hamilton	Cat no. 7653-01, 7803-04
Tamoxifen	Sigma	Cat no. T5648
NGS	Jackson Laboratory	Cat no. JAC005-000-121
DAKO mounting medium	Agilent	Cat no. S302380-2
Gelatin-coated microscope slides	FD NeuroTechnologies	Cat no. PO101

**Experimental models: Organisms/strains**		

Mouse: *Adamtsl3*^flox/flox^ BL6 strain		Both male and female P0 pups were used in the experiments.

## Materials and equipment

### Materials for mouse brain perfusion

***Note:*** The total volumes used in these recipes are suggestions and can be adjusted.

**Table:** 4% PFA Fixative

Reagent	Final concentration	Amount
PFA	4%	20 *g*
NaOH (8 N)	N/A	10 drops
Warm up to max. 60°C while stirring continuously until entirely dissolved		
0.4 M Na-phosphate buffer	0.15 M	187.5 mL
H_2_O (distilled)	N/A	Fill to 500 mL
**Total**	**N/A**	**500 mL**

Filter the solution, cool to room temperature, and adjust the pH to 7.4. Prepare 15 mL per mouse you plan to perfuse. Aliquot and store at −20°C. Aliquots should not be thawed more than once.

**CRITICAL:** Exposure to Paraformaldehyde can result in serious health effects. Handle in agreement with your institution’s biosafety committee and appropriate biosafety level.

**Table:** ACSF Stock solution (10X)

Reagent	Final concentration	Amount
NaCL	1.25 M	73.05 *g*
NaHCO_3_	260 mM	21.84 *g*/L
NaH_2_PO_4_ monohydrate	12.5 mM	1.73 *g*/L
KCl	25 mM	1.86 *g*/L
H_2_O (distilled)	N/A	Fill to 1000 mL
**Total**	**N/A**	**1000 mL**

Store at 4°C for up to 2 months.

**Table:** ACSF

Reagent	Final concentration	Amount
ACSF Stock solution (10X)	1X	100 mL
Glucose anhydr.	25 mM	4.5 *g*
1 M CaCl_2_ stock	2.5 mM	2 mL
1 M MgCl_2_ stock	2 mM	2 mL
H_2_O (distilled)	N/A	Fill to 1000 mL
**Total**	**N/A**	**1000 mL**

The solution may turn cloudy before bubbling with carbogen. Prepare fresh on the day of the experiment and adjust pH to 7.4 if necessary after it has been bubbled with carbogen for at least 30 min on ice. Prepare approximately 50 mL of ACSF per mouse you plan to perfuse.

### Materials for immunofluorescence

**Table:** Tris-Triton pH 7.4

Reagent	Final concentration	Amount
Tris	0.5 M	60.6 *g*
NaCl	1.5 M	87.7 *g*
10% Triton X-100 stock	0.05%	5 mL
H_2_O (distilled)	N/A	Fill to 1000 mL
**Total**	**N/A**	**1000 mL**

Use H_2_O to reach a volume of approximately 900 mL, then adjust pH to 7.4 using HCl before using additional H_2_O to reach a final volume of 1000 mL. Store at room temperature.

**Table:** Anti-freeze Solution

Reagent	Final concentration	Amount
0.4 M Na-phosphate buffer	31.25 mM	62.5 mL
Glucose	1 M	150 *g*
Ethylene glycol	N/A	300 mL
Sodium Azide	3.8 mM	200 mg
H_2_O (distilled)	N/A	437.5 mL
**Total**	**N/A**	**Approx. 800 mL**

Store at 4°C for up to 3 months. Brain sections in the anti-freeze solution can be stored at −20°C.

**Table:** 0.4 M Na-phosphate buffer

Reagent	Final concentration	Amount
Na_2_HPO_4_ anhydr.	324 mM	46.0 *g*
NaH_2_PO_4_ monohydr.	76 mM	10.5 *g*
H_2_O (distilled)	N/A	Fill to 1000 mL
**Total**	**N/A**	**1000 mL**

Use H_2_O to reach a volume of approximately 900 mL, then adjust pH using 1 M NaOH 1 M HCl if pH is < 7.2 and > 7.4 before using additional H_2_O to reach a final volume of 1000 mL. Store at room temperature for up to 6 months. Before use mix well by stirring.

## Step-by-step method details

### Injecting AAV into neonatal mice

**Timing: 2 h for one litter**

This step describes how to deliver virally-encoded transgenes intraventricularly into the post-natal day 0 (P0) mouse brain. We used AAV2/6-CaMKIIα-Cre-ERT2 and AAV2/8-hDlx-Cre-ERT2 to achieve widespread transduction of excitatory and inhibitory neurons in *Adamtsl3*^*flox/flox*^ mice respectively.

**Figure 1:**
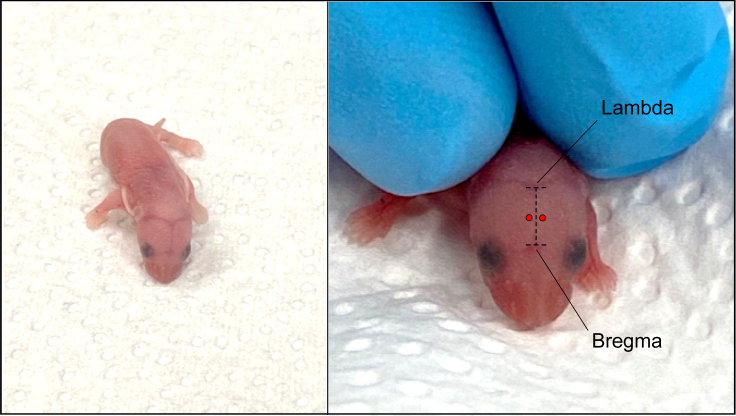
Neonatal intraventricular injection sites are indicated in red and are located between Bregma and Lambda, 1 mm from the sagittal suture
5. Place the pup on the warming pad covered with dry task wipes.
  a. Wait until it moves and appears normally.
  b. Place the pup back into the cage.
6. Repeat the procedure until all pups with AAV2/6-CaMKIIα-Cre-ERT2 have been injected.
7. Rinse the Hamilton syringe 5 times in 5 Millipore water aliquots each.
8. Load 2 μL of AAV2/8-hDlx-Cre-ERT2 into the Hamilton injection syringe and repeat the procedure until all pups have been injected.
9. Mark these pups with a laboratory pen so they can be distinguished later.

***Note:*** Regularly check and remark the pups if necessary.

1. Transfer the cage containing the mother and pups to a quiet room suitable for injection.
  a. Place a warming pad under ½ cage to keep the pups warm before injection.
  b. Cover the remaining ½ with dry task wipes (serves to warm up the pups after injection).
  c. Remove the mother from the cage and place her in a new, clean cage.
  d. Count the number of pups born and prepare aliquots of each AVV (AAV2/8-hDlx-Cre-ERT2, and AAV2/6-CaMKIIα-Cre-ERT2).
  e. Plan to inject 2 μL of AAV for 1 pup (1 μL per ventricle).
  f. Keep aliquots on ice until ready for injection.
    ***Note:*** Avoid additional freeze-thaw cycles of AAVs to conserve transduction efficacy.
    Caution: A viral titer between 5.0 × 1012 vg/mL and 1.0 × 1013 vg/mL with an injection volume of 1 μL per ventricle is typically sufficient. Adjust if necessary.
  g. Prepare 5 × 500 μL aliquots of sterile distilled water to wash the syringe before the start of the procedure. Additionally, for each AAV to be injected, prepare 5 × 500 μL aliquots of sterile distilled water to wash the syringe after injection.
  h. Prepare a 10 μL Hamilton syringe with a 32 G needle for injection by rinsing it 5 times in each of the 5 additional Millipore water aliquots.
  i. Prepare an ice bucket and place an inverted petri dish on the ice to cool.
  j. Place dry task wipes on top of it (this will be your injection stage).
    ***Note:*** Task wipes serve to protect the pup’s skin from the cold dish and serve as a flat cold surface for anesthetizing the pups.
2. Transfer one pup from the warmed cage onto the cold petri dish to induce hypothermia anesthesia.
  a. Monitor for lack of movement and after 2–3 min, confirm anesthesia by very gently squeezing a paw.
3. Gently wipe the head of the anesthetized pup with a cotton swab soaked in 70% ethanol.
  ***Note:*** Randomly select a pup from its littermates.
  Caution: Do not wait too long, as the pup's body temperature drops rapidly to ∼10°C.
  a. Load 2 μL of AAV2/6-CaMKIIα-Cre-ERT2^e^ into the Hamilton injection syringe.
    ***Note:*** Assign AAVs at random.
  b. Gently flatten the pup’s body on the task wipe and firmly stabilize the pup’s head on the neck so that the skull is facing up.
4. Identify the injection site at approximately 1 mm lateral from the sagittal suture, located between lambda and bregma.
  ***Note:*** These are visible through the skin as shown in Figure 1.
  a. Insert the needle perpendicular to the surface of the skull at the injection site to a depth of approximately 3 mm.
  b. Hold the syringe rigidly.
  c. Slowly inject 1 μL into the ventricle.
  d. Slowly withdraw the needle.
    ***Note:*** Make sure the scale is visible to monitor the volume when injecting.
    ***Note:*** If a reference is needed, mark 3 mm from the tip of the needle with a non-toxic maker.
    ***Note:*** Do not exceed 2 μL per ventricle as this may lead to ventricular enlargement and cortical thinning.
  e. Inject the contralateral ventricle using the same procedure.

10. When all pups have recovered and returned to the home cage, return the mother to the cage.
  a. Ensure that she is attending the pups before returning to the housing facility.
11. At weaning age, separate the mice and ear-punch to distinguish mice injected with AAV2/8-hDlx-Cre-ERT2 or AAV2/6-CaMKIIα-Cre-ERT2.

***Note:*** This protocol was adapted from Kim, J.-Y. et al. 2014.^4^

### Tamoxifen injection

**Timing: 30 min (for 5 days)**

In this step, Cre recombinase activity from ERT2-Cre is induced by consecutive injections of tamoxifen dissolved in corn oil. We induced Cre recombination from AAV2/6-CaMKIIα-Cre-ERT2 or AAV2/8-hDlx-Cre-ERT2 at P60 to ablate Adamtsl3 in excitatory or inhibitory neurons of adult *Adamtsl3*^*flox/flox*^ mice respectively.

12. At 2 months of age, intraperitoneally inject ½ of the mice injected with AAV2/6-CaMKIIα-Cre-ERT2 with 1 mg of tamoxifen dissolved in corn oil (10 mg/mL) to induce Cre recombinase activity from ERT2-Cre.
  a. Inject with tamoxifen for five consecutive days.
  b. Inject the other ½ with corn oil alone.
    ***Note:*** Assign mice receiving tamoxifen at random and use both sexes.
    ***Note:*** Do not exceed an injection volume of 150 μL as larger volumes can lead to pain, formation of fibrous tissue, perforation of abdominal organs, and hemorrhage.
    ***Note:*** Tamoxifen dissolution in corn oil is time-consuming. Temperatures >30°C accelerate tamoxifen dissolution in corn oil. Aliquot and store the solutions protected from light at −20°C.
  c. Repeat step 17 for mice injected with AAV2/8-hDlx-Cre-ERT2.
13. Wait for a minimum of 2 weeks after the last tamoxifen injection for efficient Cre recombination.

***Note:*** Depending on the protein of interest to be expressed or the half-life of the protein to be deleted, a longer duration may be necessary and should be evaluated prior. In general, 2 weeks is sufficient to induce complete Cre recombination from ERT2.

### Preparing mouse brains for imaging: ACSF perfusion

**Timing: 3.5 h for perfusion, followed by overnight incubation in sucrose**

This section describes a transcardiac perfusion method called “ACSF perfusion”, which prepares mouse brains for immunofluorescence labeling.

**Figure 2:**
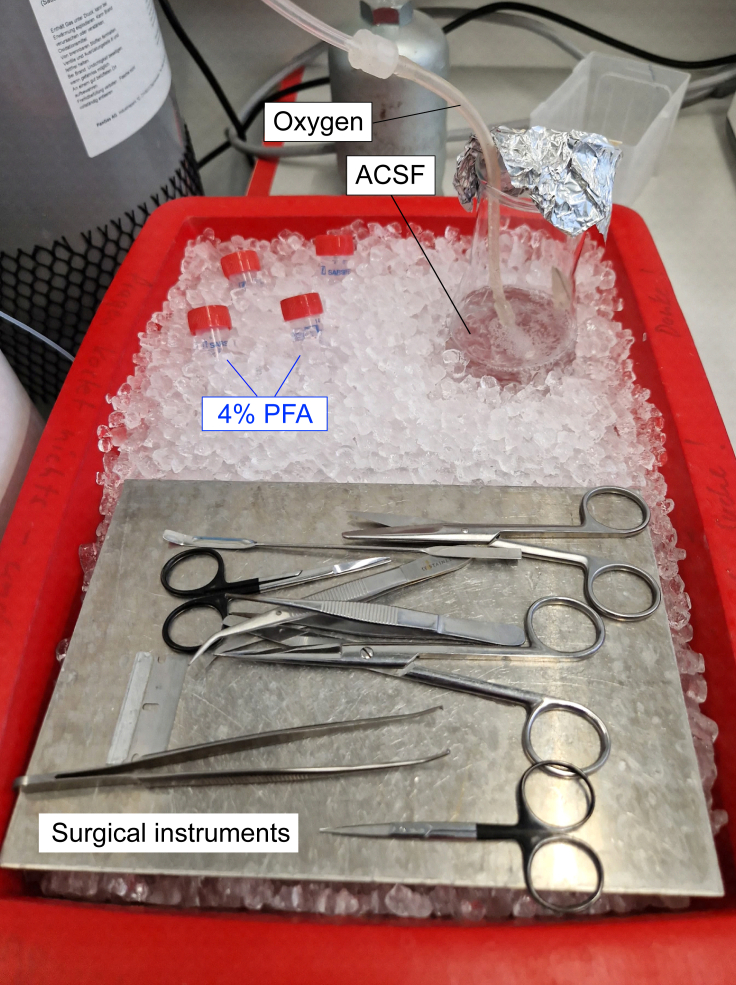
Reagents and surgical instruments required for perfusion are shown for 4% PFA fixative, oxygenated ACSF, and surgical equipment All instruments and reagents should be kept on ice.
16. Weigh one animal and anesthetize it with an overdose of pentobarbital.
  a. Check for depth of anesthesia by pinching the tail and paws.

***Note:*** An intraperitoneal dose of pentobarbital of 50 mg/kg is commonly used.

**Figure 3:**
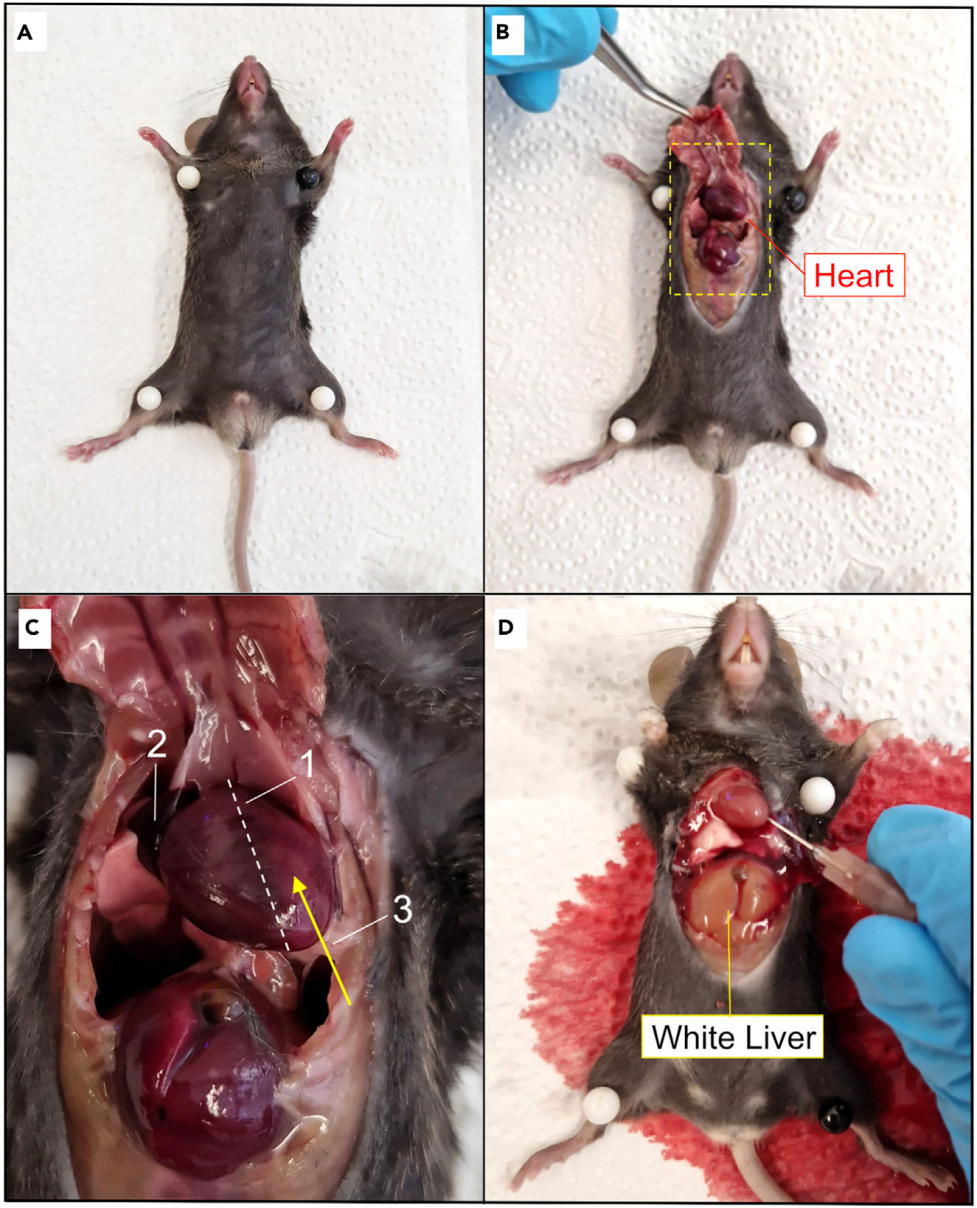
Mouse transcardial perfusion with ACSF (A) The deeply anesthetized animal is pinned on the corkboard. (B) Thorax is opened to expose the heart. (C) Reference for needle placement: 1. Axis of the heart. 2. Right atrium. 3. Needle placement. (D) Perfused mouse.
  b. Open the thorax to expose the heart and major vessels as shown in Figure 3B.
  c. Hold the heart with a pair of forceps.
  d. Insert a needle (15-gauge) through the left ventricle into the ascending aorta (follow the axis of the heart).
  e. Open the right atrium with a sharp hook as shown in Figures 3C and 3D.
  f. Start the peristaltic pump at a flow rate of up to 16 mL/min (for adult animals).
  g. When the liver turns beige-yellow, the perfusion is finished (usually less than 2 min).
  h. Stop the pump.

***Note:*** A good perfusion depends critically on the speed of the surgery. The pump should be started within 1–2 min of opening the thorax.

**Figure 4:**
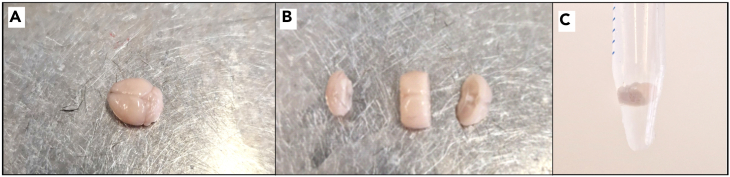
Fixation of brain tissue post-perfusion (A) Brains are rinsed in PBS, (B) cut in two or three halves, and (C) transferred into a falcon tube containing 4% PFA.
  b. Place the pieces into a falcon tube containing 15 mL of 4% PFA fixative as shown in Figure 4C. Note the time.
    ***Note:*** Do not post-fix the entire brain as this will result in insufficient penetration of the fixative.
19. After 90–120 min of post-PFA fixation, rinse the brain in cold PBS and transfer it to a falcon tube containing 30% sucrose in PBS at 4°C. Keep the post-fixation time consistent for all animals.
  a. Leave in the fridge until the brain has sunken to the bottom of the tube for cryoprotection (usually overnight).

***Note:*** To distinguish brains from tamoxifen-injected or vehicle-injected mice, mark either of them by sticking a needle through a part of the brain that is of no interest to you.

***Note:*** Do not store at 4°C for longer than 3 days due to the risk of contamination.

***Note:*** For long-term storage, snap-freeze the brains using liquid nitrogen and store them in air-tight containers at −80°C.

14. Prepare your reagents.
  a. Thaw 4% PFA fixative (15 mL per mouse) and keep on ice.
  b. Prepare 30% sucrose in PBS (15 mL per mouse) for cryoprotection and store at 4°C.
  c. Prepare ACSF from 10x stock solution.
    i. Oxygenate the ACSF solution for at least 30 min by bubbling the solution with a carbogen (95% oxygen, 5% CO2) gas mixture.
      ***Note:*** For efficient oxygenation, the end of a carbogen line should have a perforated ending, such as a plastic foam insert at the tip. This leads to the production of numerous small gas bubbles, rather than a few large ones.
    ii. Adjust the pH to 7.4 if necessary, then resume oxygenation.
15. Assemble the reagents, equipment, and surgical instruments required for perfusion as shown in Figure 2.
  ***Note:*** We recommend using the following surgical instruments: 1–2 big scissors (Sigma, Cat no. Z265993), 1–2 small micro-dissecting scissors (Sigma, Cat no. S3146), 1–2 pairs of forceps (Sigma, Cat no. F3767), a fine spatula with flat rounded ends (Sigma, Cat no. S9147) and a single edge razor blade. Equivalent dissecting tools are equally effective.
  a. Keep all solutions on ice.
  b. Keep ACSF oxygenated.
    ***Note:*** All equipment and surgical instruments used should be cleaned with 70% ethanol.

17. Perfuse the mouse transcardially with ACSF.
  a. Pin the deeply anesthetized animal on a cork board as shown in Figure 3A.

18. Remove the mouse from the corkboard, decapitate it, and remove the brain using forceps.
  a. Cut the brain into multiple pieces depending on the region of interest (sagittal or coronal) as shown in Figures 4A and 4B.
    ***Note:*** Be careful to not cut through the brain regions of interest, i.e. olfactory bulb/prefrontal cortex or hippocampal formation.

### Brain sectioning

**Timing: 2 h**

In this step, perfused and cryoprotected mouse brains are sectioned in preparation for immunofluorescence labeling. We used a sliding microtome to obtain free-floating sections, but sections can also be obtained using a cryostat and directly mounted on coated glass slides. Since the brain tissue has only been weakly fixed, the resulting brain sections are relatively fragile. It takes practice to handle these sections during cutting and staining without damaging them.

20. Set up the sliding microtome for brain sectioning.
  ***Note:*** we used a Microm HM 400 R. A cryostat may also be used. We have not tested other modes of sectioning (e.g. vibratome) for this type of tissue.
  a. Mount the blade into the blade holder and adjust it onto the microtome.
  b. Start the freezing stage on the sliding microtome.
  c. Place a drop of tissue mounting fluid on the freezing stage and wait until it is almost completely frozen.
  d. Use the razor blade to straighten the frozen mounting fluid.
  e. Place the fixed, cryoprotected brain in the desired orientation on the straightened surface of the mounting fluid.
  f. Cover immediately with mounting fluid.
  g. Wait until the mounting fluid has completely frozen.
21. Set section thickness to 40 μm (or your desired thickness) and start sectioning.
  a. Pick sections one by one with a wet brush and collect them in a multi-well plastic box filled with cold PBS (place the box on ice, using a dark background).
  b. Store the sections at 4° in PBS for up to 24 h.

***Note:*** Sections can be mounted directly onto coated glass slides.

***Note:*** For long-term storage at −20°C transfer the sections into a multi-well plastic box filled with anti-freeze solution and cover with parafilm.

### Immunofluorescence of free-floating brain sections

**Timing: 2 days**

This step involves the immunofluorescent labeling of free-floating brain sections to be subsequently visualized by confocal microscopy. Compared to the commonly used immunofluorescent labeling of fixed sections, the free-floating tissue approach allows for better antibody penetration especially when thicker sections are used for 3D reconstruction of the tissue/synapses.

**Figure 5:**
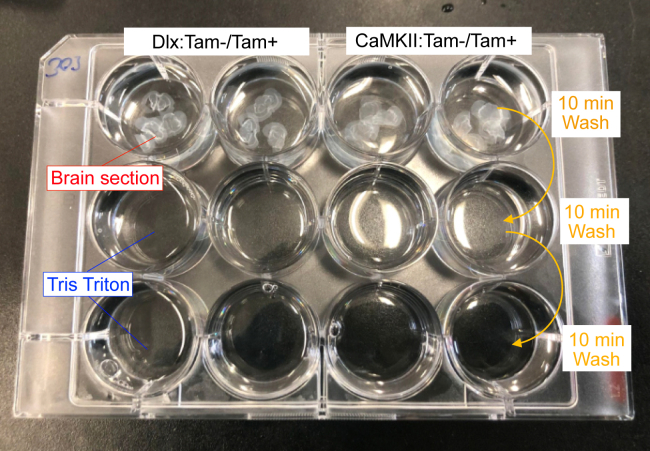
Wash brain sections using 12-well cell culture plate filled with Tris-Triton for 3 × 10 min by transferring to respective wells below
  b. Wash for 10 min on a shaker (approximately 50–100 rpm).
  c. Transfer sections to the second row and wash for 10 min on a shaker.
  d. Transfer sections to the third row and wash for 10 min on a shaker.
    ***Note:*** Take a minimum of 3 sections per mouse brain.
    ***Note:*** Mix brain sections during washing and staining that are intended for comparative analysis to control for the methodology.
  e. Prepare the primary antibody solution in Tris-Triton solution containing 2% serum (the same species as your secondary antibodies) and 0.2% Triton X-100.

22. Fill up a 12-well cell culture plate with Tris-Triton and label wells.
  a. Use a wet brush to transfer the brain sections of interest to their corresponding wells as shown in Figure 5.

**Table:** Antibody Mix example:

1° antibody solution	
**Antibody**	**Dilution**

Rabbit anti- GABA_A_R α2 subunit	1:2000
Guinea Pig anti- GABA_A_R γ2 subunit	1:2000
Mouse anti-Gephyrin	1:1000

**2° Antibody solution**	

**Antibody**	**Dilution**

Donkey anti Mouse Cy3	1:500
Donkey anti Guinea Pig Alexa Fluor 647	1:500
Donkey anti Mouse Alexa Fluor 488	1:500

***Note:*** 1° Antibody concentrations are often suggested by the manufacturer and should be tested and adjusted before starting experiments. 2° antibody concentrations are also suggested by the manufacturer.

***Note:*** The chosen secondary antibodies should be cross-adsorbed against all other species from which primary antibodies are used. Otherwise, the secondary antibodies may cross-react with incorrect primary antibodies and create false signals. In our experience, secondary antibodies from Jackson ImmunoResearch (Alexa, Cyanine, Brilliant Violet dyes) and Sigma Aldrich (CF dyes) work reliably.

***Note:*** In our experience, goat/donkey serum and secondary antibodies can be used interchangeably, as long as no primary antibody is raised in these species.

***Note:*** Labeling for Cre protein in a separate staining is necessary to confirm the neuron subtype-specific transduction of AAVs.

23. Aliquot the primary antibody solution into a labeled 9-well glass plate.

***Note:*** The maximum number of brain sections per well is usually 5–6.

***Note:*** The volume per well is usually 500 μL.

**Figure 6:**
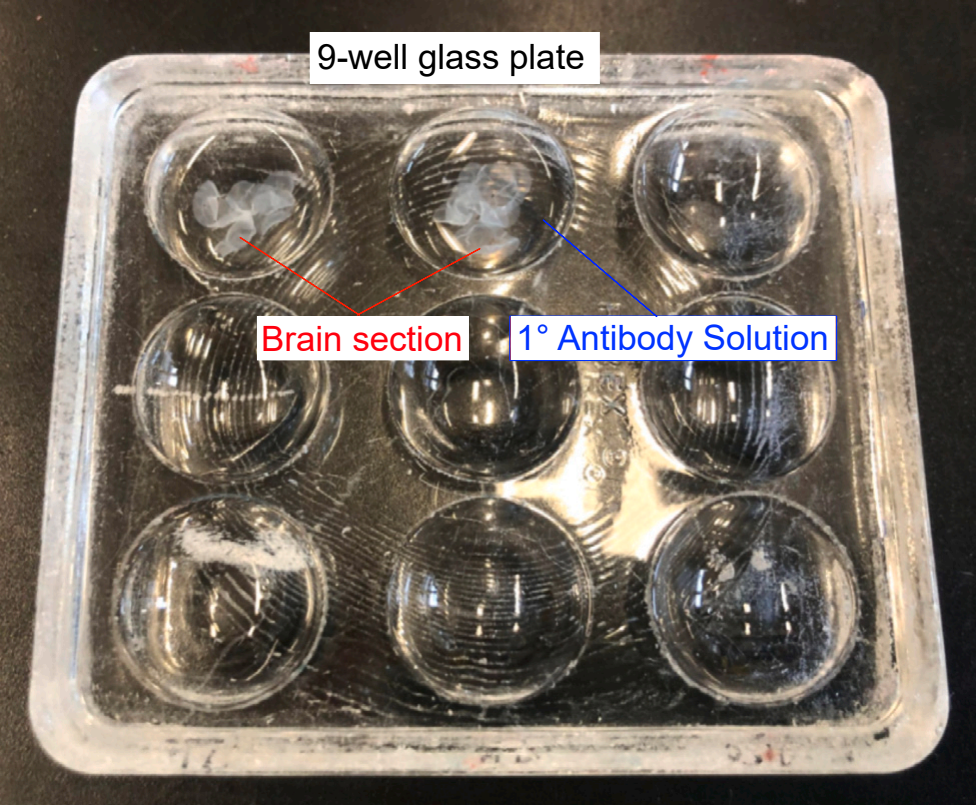
Prepare 1° Antibody in Tris-Triton solution and incubate free-floating sections using a 9-well glass plate
25. Place in a moist chamber with continuous agitation (100 rpm) overnight at 4°C.

***Note:*** Mix brain sections from different groups as you can distinguish them by the needle mark**.**

**Figure 7:**
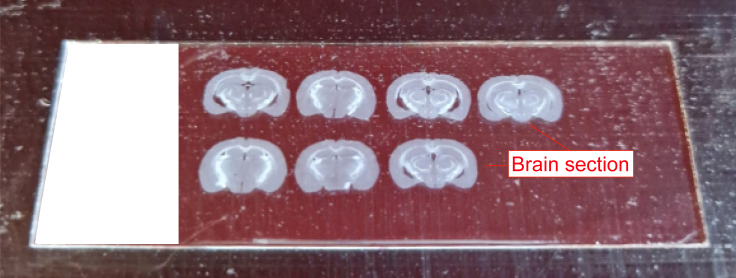
Mounted brain sections on gelatin-coated slides
  c. Note: Arrange the brain sections in an order that is sensible for your experiment.
  d. Coverslip with Dako fluorescence mounting medium.
  e. Let it polymerize at least overnight before microscopy or long-term storage.
  f. Store at 4°C in a closed slide carton.

24. Transfer the washed brain sections into the glass plate wells as shown in Figure 6.

26. Fill up and label a 12-well cell culture plate with Tris-Triton and wash sections for 3 × 10 min as in step 22.
27. Prepare the secondary antibody solution in Tris-Triton containing 2% serum.
  a. Aliquot the secondary antibody solution into a labeled 9-well glass plate as in step 22e.
28. Transfer the washed brain sections into the glass plate wells and place them in a moist chamber with continuous agitation (100 rpm) for 30 min at room temperature.
29. Fill up and label a 12-well cell culture plate with Tris-Triton and wash sections for 3 × 10 min.
  a. Label gelatin-coated slides.
    ***Note:*** the gelatin coating prevents the brain sections from sticking excessively to the glass, which can cause tearing of the tissue.
  b. Mount sections on the coated slides and let them air dry as shown in Figure 7.

### Image acquisition

**Timing: 4–6 h**

In this step, stained brain sections are imaged for analysis of synaptic puncta. ACSF perfusion followed by a short post-fixation period allows for the preservation and detection of many synaptic epitopes, which are typically damaged during PFA perfusion.

30. We performed super-resolution microscopy using an Airyscan microscope (CLSM 900 Airyscan, Carl Zeiss). Images were taken using a 63x objective to acquire near-2D slices for synaptic puncta quantification, rather than 3D imaging for the reconstruction of entire tissue blocks. The example images for the synaptic imaging are sum projections of Z-stacks (5 sections, 130 nm sectioning steps) of a 58.8 × 58.8 μm region with a pixel size of 35 nm, an optical section thickness of 260 nm, and a 2x scan average with a pixel dwell time of 0.52 μs.

***Note:*** Choose an appropriate microscope system for the detection of synaptic puncta. Regular confocal systems can be used, but super-resolution systems, such as Airyscan, are preferable.

***Note:*** Keep in mind that when comparing different brain sections from multiple animals, all imaging parameters should be kept constant including tissue focal depth (e.g. 10 μm from the top, which will minimize signal variability due to uneven antibody penetration).

***Note:*** Aim to perform the imaging for all sections within a single session to avoid inconsistent imaging across multiple sessions.

31. Switch on the microscope system and let it run until it has reached a stable temperature.
  a. Perform any necessary calibrations and other startup procedures to ensure consistent system performance during image acquisition.

***Note:*** The minimum duration between turning the system on and imaging, as well as necessary calibrations, are provided by the manufacturer.

32. Remove the slide to be imaged from the slide carton and let it warm up to room temperature for a few minutes.
  a. Clean the slide and place it on the microscope.
33. If available, use a low-magnification objective and a ubiquitous marker (such as DAPI or NEUN) to locate the brain region to be imaged.
  a. Switch to a high-magnification objective for imaging. This will typically be an oil immersion objective.

***Note:*** Do not excite the synaptic channels you wish to analyze when using the eyepiece for locating the target region. Exciting these channels unnecessarily may cause photobleaching and falsify the results.

34. Locate a suitable imaging location within the target region to determine the optimal acquisition settings.
  ***Note:*** This particular location should not be used during the final imaging, as it will experience some bleaching during testing.
  a. Follow your system’s procedure to set up the optimal image resolution, scanning speed, and different color channels you wish to image.
    ***Note:*** These settings should ensure that there is no bleed-through between the different channels.
    ***Note:*** For the analysis of near-2D images, large Z-stacks should be avoided. Generally, we recommend either taking a single image, or a Z-stack no larger than 1 μm in depth.
    ***Note:*** For Airyscan imaging, it is recommended to take a Z-stack with at least 5 optical sections with optimal spacing between them, as determined by the software. This will enable the “3D-processing” option in the Airyscan processing tab, which is significantly better than the regular 2D Airyscan processing.
  b. Set the laser intensity and detector gain for each color channel individually.
    ***Note:*** Ensure that there are no saturated pixels in the final image.
  c. Using these settings, perform imaging of your target region.
    ***Note:*** It may be helpful to take a high number of images (6–10) of the first brain section and analyze them to get an idea of the image-to-image variability within one specimen. The observed variability can then be used to choose a suitable number of images to take for each subsequent section.

## Expected outcomes

**Figure 8:**
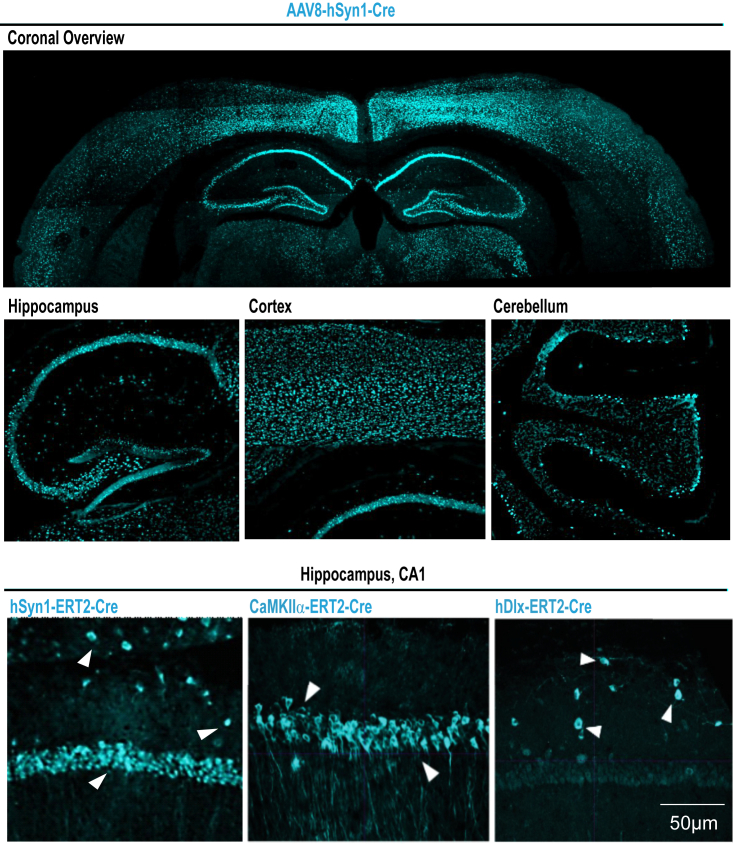
Transgene expression across brain regions and synapse-specific after AAV lateral ventricle P0 injection Top:Coronal section obtained from P0 pups injected with AAV8-hSyn1-Cre at 3 months stained for Cre protein. Below: Sagittal sections obtained from P0 pups injected with AAV8-hSyn1-Cre at 3 month stained for Cre protein focusing on the hippocampus, cortex, and cerebellum. Bottom: Hippocampal coronal section obtained from P0 pups injected with AAV8-hSyn1-ERT2-Cre, AAV6-CaMKIIα -ERT2-Cre or AAV8-hDlx-ERT2-Cre at 3 month (Cre-recombination induced at 2 month) stained for Cre protein focusing on the CA1 region.

The expected outcome of delivering genetically engineered AAVs by neonatal intraventricular injection is the widespread expression of transgenes. For visualization, we obtained coronal and sagittal sections from a 3-month-old mouse injected at P0 with AAV8/2-hSyn1-Cre shown in Figure 8 (Top); long-lasting detection of Cre protein across brain regions highlights the efficiency of utilizing neonatal intraventricular injection for AAV delivery. Of note, cortical and hippocampal brain regions tend to exhibit consistently high expression of transgene, while dorsal brain regions such as the cerebellum tend to exhibit a more variable rate of transduction as shown in Figure 8 (Middle). To investigate the function of pre- or post-synaptically localized proteins in the hippocampal CA1 region, we utilized a combination of AAVs: AAV6/2-CaMKIIα-Cre-ERT2 and AAV8/2-hDlx-Cre-ERT2 (AAV8/2-hSyn1-Cre-ERT2 as control). Following Cre recombination at 2 months, this combination enables the >90% of the targeted cells exhibit deletion of our protein of interest, Adamtsl3, in the adult brain and the investigation of presynaptic (hDlx-Cre-ERT2) and postsynaptic (CaMKIIα-Cre-ERT2) Adamtsl3 function at GABAergic synapses at CA1 pyramidal cell somata (Figure 8 Bottom).

**Figure 9:**
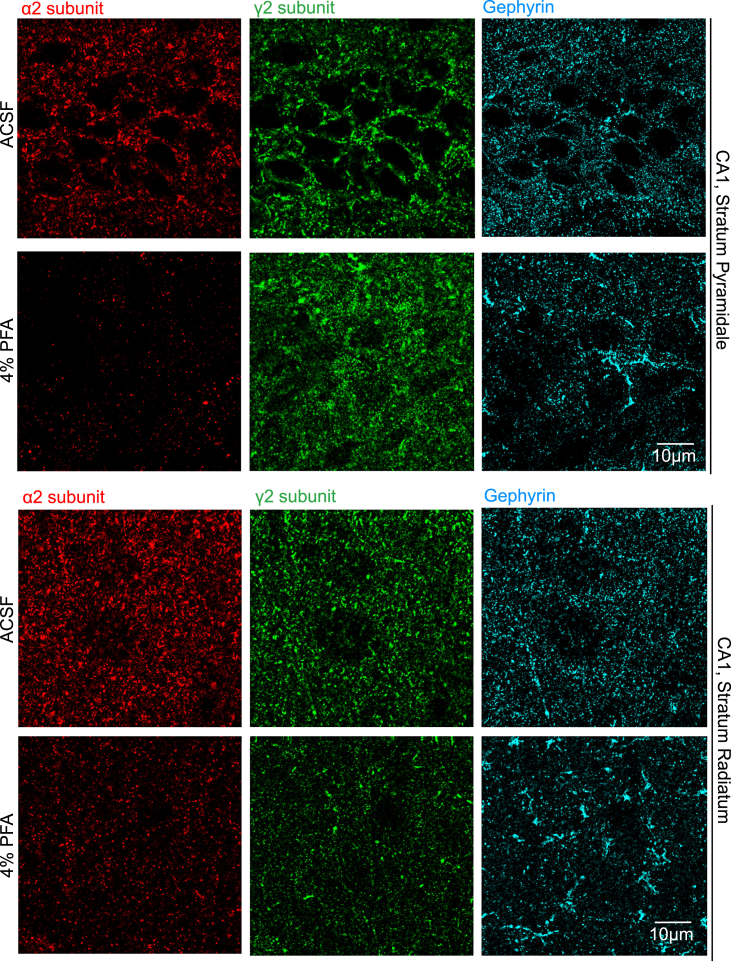
Inhibitory synapse analysis between ACSF and PFA perfused brain tissue Top:lmmunofluorescence in the CA1 Stratum pyramidale of sections obtained from ACSF (top) and PFA (bottom) perfused brains. GABA_A_ receptor subunit α2 (red), GABA_A_ receptor subunit γ2 (green), Gephyrin (cyan). Bottom. Immunofluorescence in the CA1 Stratum radiatum of sections obtained from ACSF (top) and PFA (bottom) perfused brains. GABA_A_ receptor subunit α2 (red), GABA_A_ receptor subunit γ2 (green), Gephyrin (cyan).

**Figure 10:**
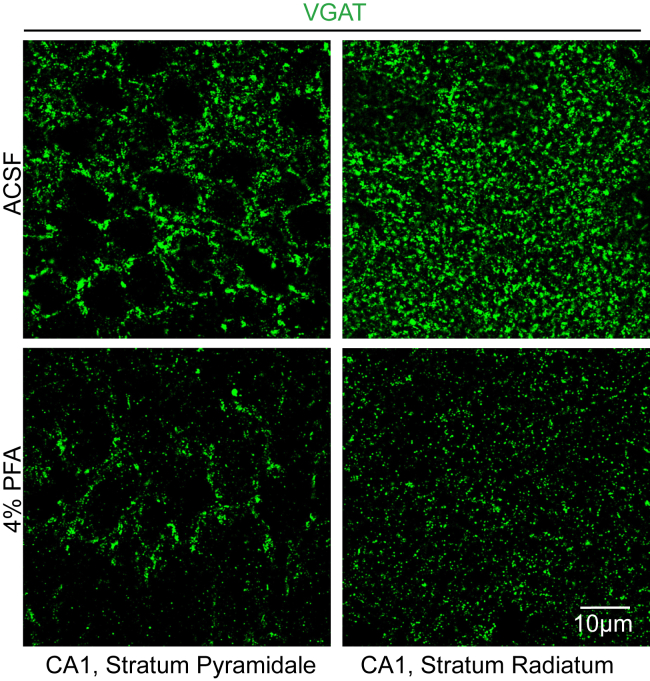
Staining for VGAT (green) in the CA1 Stratum pyramidale and Stratum radiatum of sections obtained from ACSF or PFA perfused brains

The expected outcome of the described ACSF perfusion and immunofluorescence staining protocol is the superior detection of synaptic proteins compared to PFA-perfused sections. For comparative visualization, we also performed the immunofluorescence staining protocol on PFA-perfused brain sections (Perfusion with PBS and 4% PFA before an additional 3-h post-fixation). The resultant images shown in Figures 9 and 10 were taken in the *Stratum Pyramidale* and *Stratum Radiatum* as described in the section “Image Acquisition”. Images obtained from ACSF and PFA perfused sections were subsequently processed as Z-stack sum-projection modality using identical background subtraction and brightness/contrast settings in the FIJI software (ImageJ). Under identical image processing parameters, the superior signal/noise ratio and spatial resolution of synaptic protein markers in ACSF perfused brain sections are highly visible. Effective labeling as shown is crucial for understanding the function of synaptic proteins; in combination with AAV-mediated segregated manipulation of pre-synaptic or post-synaptic neurons, this protocol thus enables the reliable investigation of pre- or post-synaptically localized proteins, as well as the site-specific function of secreted proteins localized in the synaptic cleft.

## Limitations

Our protocol describes the methods for neonatal injection of AAV, ACSF perfusion, and immunofluorescence labeling in the hippocampal CA1 region. AAV selection as well as antibody selection was therefore performed based on the region of interest and should thus not be used to study other brain regions/synapses. While AAV spread to hippocampal, thalamic, and cortical regions is high, spread to more anterior/posterior regions is variable and should be considered before starting the experiment. Another limiting factor is the proportion of mice lost due to lack of maternal care (varies by strain and breeding pair) following neonatal injection (loss of mice to injection is negligible), which is why a large number of pups is needed to meet relevant n numbers for statistical comparisons.

## Troubleshooting

### Problem 1: Low transduction efficiency

High AAV transduction efficiency is essential to achieve widespread transgene expression. Transduction efficiency is influenced by many factors, including the precision of P0 injection and AAV titer (Step 1).

### Potential solution

To achieve a high transduction efficiency, it is of utmost importance to use high AAV titers and correctly insert the needle at the injection site (location and depth). Only when AAVs are injected into the ventricles, they can move freely from the ventricles into the brain and spread. Therefore, you must practice the injection multiple times in advance to ensure that the injection site is hit correctly, that you are maintaining a 90-degree angle to the surface of the skull, and are not moving the needle up while injecting (common). Additionally, keep your arms firmly planted on a steady surface to minimize any unwanted movement. To maintain AAV titer, avoid freeze-thaw cycles by aliquoting AVVs.

### Problem 2: Off-target expression or expression in the absence of tamoxifen induction

The usefulness of tamoxifen-inducible transgene expression depends on its faithful expression. Any expression in cell types other than the desired one, as well as the expression in the correct cell population at the wrong time, can confound results (Step 2).

### Potential solution

When selecting an AAV, choose one that has been validated, either in-house or in the literature. Even though well-known promoters such as human Synapsin 1 (hSyn1) or CaMKIIα have been used extensively, it is essential to validate their expression. Validation should occur prior to recombination to evaluate tamoxifen-independent transgene expression and post-recombination using immunofluorescent markers for the transgene and desired cell type. Because tamoxifen-independent recombination has been reported, it is additionally important that your control cohort is AAV injected but did not receive tamoxifen. When your AAV shows off-target expression and high expression in the absence of tamoxifen induction, choose a different one.

### Problem 3: Low Cre recombination

The effectiveness of tamoxifen injection is essential to obtain reliable transgene recombination (Step 2).

### Potential solution

Make sure you are performing the intraperitoneal injections correctly as bleeding at the injection site, peritonitis or injection into the gastrointestinal tract/bladder reduces the uptake of tamoxifen. Additionally, make sure you keep the tamoxifen cool (4°C) for the duration of injections and always keep it in a light-blocking container or wrapped in foil as tamoxifen is highly light-sensitive.

### Problem 4: Poor transcardiac perfusion

Proficient perfusion is vital for the subsequent labeling of synaptic markers using immunofluorescence and is usually visible during perfusion by the liver turning yellowish-white and after the perfusion by a white brain (see Step 3).

### Potential solution

There are several indicators of a faulty perfusion. Perfusion solution coming out from an animal’s nostrils or mouth, for example, indicates that you have inserted the needle incorrectly and that the solution is flowing into the lungs. Practice to properly insert the blunted perfusion needle into the left ventricle by following the axis of the heart. Also, be careful to not enter the needle too deep, as this may lead to the internal wall of the heart rupturing. If you do accidentally puncture the heart, try clamping the puncture while you continue to perfuse. If necessary, remove and reposition the needle to try again. If all attempts are unsuccessful, you may not be able to use the animal for continuous experimentation, which is why practicing perfusion beforehand is very important.

### Problem 5: Low detection of immunofluorescent-labeled proteins

Reliable immunofluorescence labeling of synaptic markers is essential for subsequent image acquisition and analysis (Steps 4–10). Weak or no immunofluorescent signals can be caused by many factors including antibody incompatibility or permeabilization method.

### Potential solution

When you prepare for your immunofluorescence experiments, it is crucial to verify that both primary and secondary antibodies are compatible to avoid cross-reactivity. Although manufacturers often provide recommended dilutions and incubation times, test these in advance. For some antibodies, increased permeabilized may be necessary and should be adjusted if necessary (see Step 34; 0.4% Triton X-100 increases the penetration of antibodies). The primary and secondary incubation times may be additionally adjusted. There is often a balance regarding incubation time between tissue penetration and background signal. It may be useful to incubate secondary antibodies against ubiquitous targets (e.g., NEUN, CAMK2A, or MAP2) overnight at 4°C to enhance penetration depth, and then the secondary antibodies against synaptic markers for 30 min at room temperature to maintain a low background signal. To mitigate excessive background fluorescence, adjust the antibody concentration and enhance the washing steps to 3 × 15–20 min (see Steps 4 and 5). It is also important to incubate secondary antibodies and store samples in a dark environment to prevent the fading of fluorophore signals, which can occur with prolonged light exposure (Steps 6–10). Another important consideration is the selection of bright, photostable dyes for your secondary antibodies. Some dyes are not bright enough for the detection of low-abundance synaptic proteins. In our experience, the following dyes can be used to detect synaptic markers: Brilliant Violet 421, Alexa 488, Cy3, Alexa 568, CF568, Alexa 647, and Cy5. Other dyes may work as well, but any dye combination should be verified in a test staining for signal quality and lack of spectral overlap.

## Resource availability

### Lead contact

Further information and requests for resources and reagents should be directed to and will be fulfilled by the lead contact, Shiva K.Tyagarajan (shiva.tyagarajan@gmail.com).

### Technical contact

Technical questions on executing this protocol should be directed to and will be answered by the technical contacts, Teresa Cramer (crame676@mit.edu) and Andrin Abegg (andrin.abegg@pharma.uzh.ch).

### Materials availability

All reagents generated in this study are available from the lead contact without restriction.

### Data and code availability

• All data reported in this paper will be shared by the lead contact upon request.
• Any additional information required to utilize the data reported in this paper is available from the technical contact upon request.
